# Naturalistic face adaptation: How we adapt to freckles fast and sustainably

**DOI:** 10.1177/20416695231195262

**Published:** 2023-09-16

**Authors:** Sandra Utz, Ronja Mueller, Tilo Strobach, Claus-Christian Carbon

**Affiliations:** Department of General Psychology and Methodology, 14310 University of Bamberg, Bamberg, Germany; Bamberg Graduate School of Affective and Cognitive Sciences (BaGrACS), University of Bamberg, Bamberg, Germany; Research Group EPÆG (Ergonomics, Psychological Æsthetics, Gestalt), Bamberg, Germany; Department of Psychology, Institute for Cognitive and Affective Neuroscience (ICAN), Medical School Hamburg, Hamburg, Germany; Department of Psychology, Institute for Cognitive and Affective Neuroscience (ICAN), Medical School Hamburg, Hamburg, Germany; Department of General Psychology and Methodology, 14310 University of Bamberg, Bamberg, Germany; Bamberg Graduate School of Affective and Cognitive Sciences (BaGrACS), University of Bamberg, Bamberg, Germany; Research Group EPÆG (Ergonomics, Psychological Æsthetics, Gestalt), Bamberg, Germany

**Keywords:** face adaptation, freckles, naturalistic, perception, memory, non-configural face information

## Abstract

While sunbathing, our skin becomes susceptible to quite remarkable changes in visual appearance, that is, freckles appear or increase in intensity—most obviously on the face. Research on face adaptation repeatedly showed that the inspection of manipulated versions of faces (so-called adaptor faces) leads to robust and sustainable changes in the perception of subsequently presented faces. Therefore, during the adaptation phase of the present study, participants saw faces with either strongly increased or decreased intensities of freckles. After a 5-minute break, during the test phase, participants had to identify the veridical (non-manipulated) face out of two faces (a slightly manipulated face combined with a non-manipulated face). Results showed strong adaptation effects to increased and decreased levels of freckles. We conclude that updating facial representations in memory is relatively fast, and these representation updates seem to sustain over a certain time span (at least 5 minutes). Face-specificity of our effects will be discussed. The results align with our everyday experience that the appearance of freckles in spring is a salient change in a familiar face; however, we seem to not register these changes after a few exposures due to a loss of information quality.

The early spring sun considerably changes the appearance of faces: not only do faces start to get tanned, but also freckles begin to spread all over some faces. With melanocytes overproducing melanin granules, freckles become darker and more visible in the light. However, after a few exposures, people surprisingly quickly adapt to these new facial features in otherwise quite familiar faces. The same face without freckles would now be perceived as almost “unnatural”, and other faces with freckles are entirely “natural.” This makes freckles an interesting case of dynamically and naturally changing face properties (through exposure to sunshine) along with complexion (typically comprising color and texture of the skin, especially on the face; Utz et al., 2023) and other more linear changes such as aging effects (wrinkles, skin quality, and macules).

Adaptation (after)effects are perceptual distortions resulting from extended periods of visual exposure to almost every kind of stimulus (Köhler & Wallach, 1944; Leopold et al., 2001). Since we seem to be able to adapt to any kind of stimulus, it seems worth having a closer look at our ability to adapt to something that we are confronted with in typical everyday-life contexts. Experiments investigating our ability to adapt to artificial manipulations or distortions in faces—so-called face adaptation effects (Webster & MacLin, 1999)—have repeatedly shown that inspecting manipulated faces (so-called adaptor faces) leads to changes in the perception of subsequently presented faces. Typically, original or baseline images are then perceived as manipulated in the opposite direction of the adaptor, while images that are more similar to the adaptor are perceived as normal. It has been found that these effects are not just restricted to the identity of faces, but they also take into account attractiveness, age, etc. Face adaptation effects have been found to be highly robust and sustainable (for an overview, see Mueller et al., 2020; Strobach & Carbon, 2013; Webster & MacLeod, 2011). For instance, Carbon, Strobach, et al. (2007) and Carbon and Ditye (2011) showed adaptation effects to configurally distorted celebrity faces that lasted one day and even up to one week, respectively. From a general perspective, those adaptation effects align with Gestalt theories, such as Helson's adaptation level theory (Helson, 1947, 1964). This theory states that perception is not only a function of the present stimulus and a mere mediating process between a single stimulus and its corresponding sensation but also a function of past stimuli; responses are dependent on the state of adaptation of an organism to all such stimuli. Individuals strive to put all the stimuli (such as faces) they perceive in a meaningful order by describing them by a scale value on relevant dimensions of face information, thereby being able to make comparisons with other faces. Thus, faces are represented by zero points at the middle point of bipolar dimensions relevant for identification, and these faces are perceived as neutral, representing the central anchor face stimulus. These zero points are shifted towards the adaptor stimulus, and the identification of a face is changed subsequently.

These adaptation effects were not restricted to faces presented during adaptation but could also be transferred to faces not previously seen, such as different images of the same identity or even different identities (Carbon & Ditye, 2011, 2012; Carbon, Strobach, et al., 2007; Fox & Barton, 2007; Strobach et al., 2011; Webster et al., 2004). This indicates that these adaptation effects are based on more abstract and structural processes than pictorially-oriented processes. Since these effects are robust and flexible concerning their sustainability and level of abstraction, adaptation seems to be caused neither by aftereffects located on a retinal level nor by simple episodic memory processes such as the recency effect (a cognitive bias or tendency to remember most-recently-presented information best). It seems more likely that such adaptation effects are based on higher cognitive processes, driving the permanent update of our mental face representations. Based on this theoretical processing framework, we could explain face adaptation in the following way (following, e.g., Carbon & Ditye, 2011, 2012; Ditye et al., 2013; Mueller et al., 2020, 2021a, 2021b): if we are inspecting a strongly manipulated familiar face (the adaptor face), our internal memory representation is immediately and automatically updated, integrating the newly inspected face exemplar. If we have to identify the original or baseline face out of several faces afterwards (in experimental adaptation paradigms, participants usually have to indicate the original or baseline face presented with a slightly manipulated familiar face), we have to compare the presented face with our—now updated—mental representation. For example, if someone adapted to a face with increased brightness, a face shown afterwards with only slightly increased brightness (and therefore more similar to the adaptor face) is then perceived as being more veridical (i.e., representing the original or baseline face) than the actual original or baseline face. The original or baseline face itself will be perceived as slightly manipulated in the opposite direction to the adaptor face, that is, it will appear to have decreased brightness (Mueller et al., 2020, 2021a). Finally, Ditye et al. (2013) showed that sleep facilitates face adaptation, which is another hint that facial adaptation might be a memory-related effect. Several results demonstrate remarkably long-lasting adaptation effects (e.g., Carbon & Ditye, 2011; Carbon, Strobach, et al., 2007) and argue in the direction of a rather higher cognitive mechanism of these effects, that is, a memory-based effect (and against a purely retinotopic effect).

When investigating adaptation effects, most studies so far have used manipulations of configural information in faces (i.e., information related to spatial relations or 2^nd^-order relations; see review by Mueller et al., 2020; Strobach & Carbon, 2013). Face research, however, also tells us that other facial features play a role in face processing, for instance, local feature information such as the width of the nose or the size of the mouth (e.g., Macho & Leder, 1998), texture (e.g., Liu et al., 2005), or color (e.g., Lee & Perrett, 1997). Rakover and Teucher (1997) even assumed that 91% of facial recognition is based on its features. From prosopagnosia research, we also have indications that featural information might be the basic information we use for processing faces (Carbon, Grüter, et al., 2007). Moreover, featural (Carbon & Leder, 2005a) as well as categorical information based on single features (Carbon et al., 2018) are processed very early in the process of face recognition—much earlier than more configurally-based information.

One of the few research attempts investigating adaptation effects based on configural in comparison to non-configural face information is the study by Yamashita et al. (2005). They primarily investigated face adaptation effects to configural distortions but were also interested in exploring the selectivity of these effects to changes in varied facial information (configural and non-configural information, such as color). Although some facial information seems to have a more significant impact on adaptation effects (e.g., color) than other information (e.g., contrast), all changes in varied facial information lead to weaker adaptation effects when altered. The authors, therefore, revealed that both configural and non-configural face information contribute to effective face processing, but the effects cannot clearly be attributed to either configural or non-configural information since they were only investigated in combination. The studies by Mueller et al. (2021a, 2021b) are, therefore (to the authors’ knowledge), the first studies systematically implementing only manipulations to non-configural color face information within an adaptation paradigm. Using familiar celebrity faces manipulated in terms of brightness or saturation, these studies could reveal that non-configural face information also leads to relatively robust adaptation effects. Therefore, they could support the assumption that non-configural face information is not only relevant in the perception of faces but also plays an important role in the storage of faces. However, those intense brightness or saturation manipulations to familiar faces might appear rather irritating or unnatural and leave the question unanswered regarding whether those adaptation effects would be similar in pre-experimentally unfamiliar faces, where more natural changes were applied.

## The Present Study

Since face adaptation research mainly focuses on configural information, the present study tries to extend knowledge regarding non-configural face information due to its importance in several face processing and memory theories. We used more ecologically valid material by testing typical adaptation effects regarding quite natural changes in faces, that is, changes in the intensity of freckles. Freckles were experimentally varied and not selected from a natural selection in order to optimize the controllability of stimulus intensities. We decided to employ pre-experimentally unfamiliar faces (face adaptation studies normally use famous faces) to decrease potential conflicts with the already-established representation of faces because participants had no information about whether these pre-experimentally unfamiliar faces had freckles or not. In a learn-then-test paradigm, participants were familiarized with the non-manipulated faces (all faces showed freckles) and then, during the adaptation phases, exposed to faces with strongly decreased and strongly increased intensities of freckles or with non-manipulated face versions. During the following test phase, participants had to choose in a two-alternative-forced-choice (2-AFC) task the veridical face out of two presented faces (the BASELINE face and a slightly manipulated one). Using a paradigm that separated the adaptation and test phase by approximately 5 minutes (see Carbon, Strobach, et al., 2007; Mueller et al., 2021a, 2021b), we ensured that any effects were not merely based on ultra-short-term (retinotopic) perceptual effects. Since we also intended to determine whether possible effects are transferable, we employed different images of the same person (structural transfer) or completely different identities (cross-identity transfer) in the adaptation and test phases.

## Method

### Participants

Thirty-six participants (26 female, 10 male) aged 18 to 32 years (*M*_age_* *= 21.4 years; *SD*_age_ = 3.7) were tested. This number of participants was calculated a priori via power analysis (Faul et al., 2007) based on a mixed-design analysis of variance (ANOVA) with a 3 (between-subjects) × 3 (within-subjects) factor design being able to detect a medium effect size *f* of 0.25 (Cohen, 1988) given an *α* = .05 and a test power (1 - *β*) = .80. Similarly sized effects for the factor adaptation (the primary focus in our study) were revealed in previous studies (e.g., Carbon & Ditye, 2012: $\eta_{p}^{2}=.316$ —medium effect; Carbon & Ditye, 2011: $\eta_{p}^{2}=.350$ —medium-to-large effect). All participants had normal or corrected-to-normal vision (assessed by a standard Snellen eye chart test) and normal color vision (assessed by a short version of the Ishihara color test). All participants were White Europeans and undergraduates at the University of Bamberg. They received course credit points for their participation or were paid €15. They had no prior experience with the present task and were naïve to the purpose of this experiment. The study was conducted according to the principles expressed in the Declaration of Helsinki and the ethical principles of the German Research Foundation (Deutsche Forschungsgemeinschaft [DFG]) and the Association of German Professional Psychologists (Bundesverband Deutscher Psycholog*innen [BDP]). Each participant gave written informed consent, and the details and rationale of the study were discussed with each of them upon completion of the experiment.

### Apparatus

Participants were seated approximately 60 cm in front of a 61.2-cm EIZO ColorEdgeCG245W-LC monitor running at a screen resolution of 1,920 × 1,200 pixels with a refresh rate of 60 Hz controlled by a standard PC. They responded to the experimental task by pressing response keys on a QWERTZ keyboard. Stimuli, trials, and experimental blocks were created with the up-to-date version of RStudio (1.3.1093), ensuring high precision in executing the correct timing of the study.

### Stimuli

Faces were chosen from the Chicago Face Database (Version 2.0.3). All photographs fulfilled the following criteria: full face in frontal view, straight gaze, no facial features covered with hair, no freckles, and no glasses. These photographs were presented at a size of approximately 210 × 288 pixels in the experimental phases.

**Figure 1.**
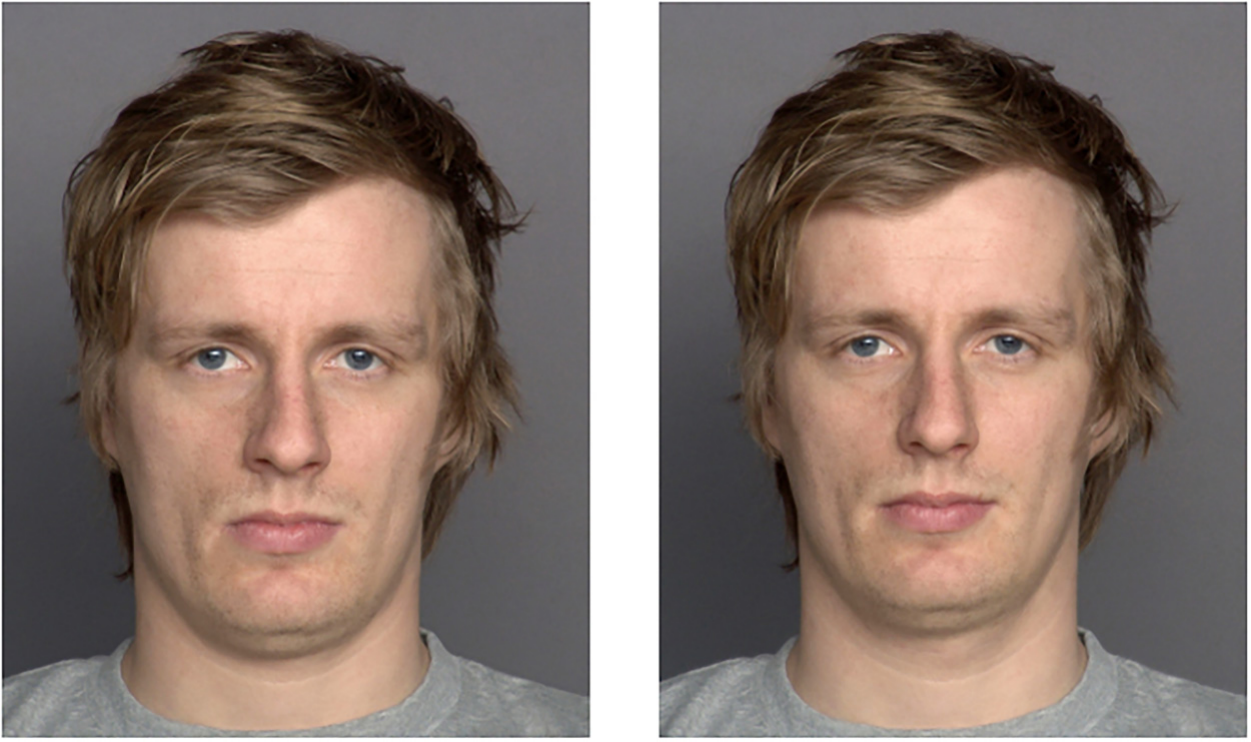
Example for picture A and picture B of the same person.

Eighteen faces (9 male, 9 female) were randomly assigned to three different sets of faces (I, II, III), containing six faces each. Two color photographs (A, B) of the same person, that is, two different depictions of the same identity, were selected, resulting in six different stimulus sets necessary to realize the three different transfer levels. The transfer levels varied in the amount of overlapping information between images presented in the adaptation and test phases. On the *pictorial* transfer level, stimuli in the adaptation and test phases are pictorially identical (e.g., presenting Picture A of a person as an adaptor and test stimulus [= the same photo of the same identity]). On the *structural* transfer level, the same identity is used in the adaptation and test phases, employing a different depiction (e.g., presenting Picture A of a person as an adaptor and Picture B of the same person as a test stimulus [= different photos of the same identity]; see Figure 1). This was possible since we had two different depictions of each identity. On the *cross-identity* transfer level, different identities are presented in the adaptation and test phases (e.g., Picture A of a person as an adaptor and Picture A of another person as a test stimulus [= different photos of different identities]).

**Figure 2.**
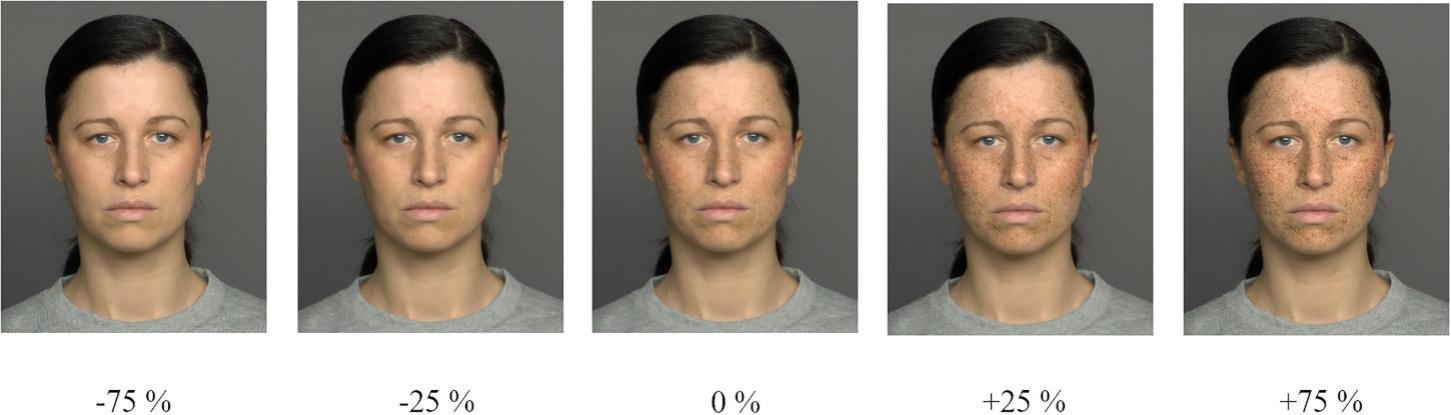
Example for the different freckle intensities ranging from -75% to +75%. The middle intensity (0%) was used as the BASELINE picture for the learning phase, extreme intensities (-75% = MINUS EXTREME, +75% = PLUS EXTREME) were used for the adaptation phase, and middle intensities (-25%, +25%) were used in combination with the BASELINE picture (0% = BASELINE) for the test phase.

The salience of freckles was manipulated through changes in intensity and number (density) of freckles using Adobe Photoshop CC (Version 19.0). Note that the facial background was kept constant. We carefully manipulated the pictures to make them look as realistic as possible, even at the extreme levels presented in the adaptation phase of the experiment. We created for each picture a series of variants from -75% up to +75% with increasing salience of freckles in equal 25% steps, starting from the faces with middle intensity (0% = BASELINE). This creation resulted in seven different variants per base picture ([1]: −75% = MINUS EXTREME, [2]: −50%, [3]: −25% = MINUS, [4]: 0% = BASELINE, [5]: +25% = PLUS, [6]: +50%, [7]: +75% = PLUS EXTREME). In more detail, we changed the combination of number, size, intensity (saturation), and opacity of the freckles by 25% steps (referring to an increase or decrease in freckle intensity by 25%). Through pilot testing showing that -/+50% variants were too far away from the expected adapted versions, we only used -/+25% variants as test versions (tested against the BASELINE versions = 0%), while the -75% and +75% extreme variants were employed as stimuli for the adaptation phase (the so-called adaptors). The resulting five different variants can be seen in Figure 2 for one exemplary stimulus series.

### Procedure

The study consisted of four parts: a learning phase, an adaptation phase, a 5-minute reading break, and a test phase.

#### Learning Phase

Participants’ task was to become familiar with the unknown faces and correctly name them. To do so, they had to learn the names we randomly assigned to the depicted people. During the learning phase, faces of the middle intensity of freckles (0%, see Figure 2) were presented (i.e., the BASELINE faces of the test phase). For each trial, one face of a total of 12 faces (six male, six female) from two sets of stimuli (depending on the experimental version of the adaptation phase they were assigned to) was presented centrally. During learning, faces and names were presented together until participants pressed the space bar for the next face and name. After the 12 faces and names were presented, participants’ familiarity with the faces and respective names was tested. This was done by presenting each face again, for which participants had to enter the correct names via the keyboard. After entering all the names twice in a row correctly, they had to further accomplish an additional test where the same procedure was used but inverted faces were displayed. This was done to maximize the (featural and configural) elaboration of faces. Whenever participants made mistakes, an entirely new learning sequence started. Note that the purpose of the whole learning phase was to get familiar with the faces in a reasonable manner. Therefore, participants were not tested on correctly naming those faces again in the course of the study. They were, however, told to keep those faces in mind since they would need them for the later test phase.

#### Adaptation Phase

After the successful completion of the learning phase, the adaptation phase started. The same faces from the learning phase were presented as adaptors, but in three versions: either originally presented (BASELINE), with strongly increased freckles (+75%; PLUS EXTREME) or strongly decreased freckles (−75%; MINUS EXTREME). Participants were randomly assigned to one of the adaptation conditions (between-participants factor). To control for retinal effects, faces were presented at six different monitor positions (top-left, top-center, top-right, bottom-left, bottom-center, and bottom-right). To increase inspection times while avoiding fatigue effects, the presentation time of adaptors was varied (2, 3, or 4 seconds; adapted from Carbon, Strobach, et al., 2007). Positions and inspection times were randomized throughout the experiment. Each adaptor was presented 18 times (three presentation times × six positions). Each participant adapted to two sets of faces (same as in the learning phase), that is, pictures A or B of two (out of the three) sets of faces (I, II, III). One set of faces was not shown during adaptation. Participants’ task was to decide whether the depicted person was male (by pressing the key “A”) or female (by pressing the key “L”). A total of 216 trials were presented (12 faces × six positions × three presentation times). After half of the trials (i.e., 108 trials), participants were allowed to take a break and were free to decide when they wanted to start the second half of the adaptation phase.

#### 5-Minute Break

During the break in between the adaptation and test phases, participants’ task was to read a completely unrelated (to the current experiment) text about the geography of the Republic of Kenya (source: https://www.lernhelfer.de/schuelerlexikon/geografie/artikel/republik-kenia#). The reading task aimed to prevent the mental recall of the previously seen faces. After 5 minutes of reading, participants were interrupted by the experimenter and asked to enter the test phase.

#### Test Phase

Participants were instructed that they had to do a 2-AFC task where two different versions of the same picture were presented. Participants had to decide if the left or the right of the presented pictures represented the BASELINE picture by pressing “A” for the left or “L” for the right picture. Participants were clearly instructed what we meant by “BASELINE” pictures, that is, that they had to identify the pictures they were made familiar with during the learning phase.^1^ After the instruction, participants could start the test phase by pressing the space bar on the keyboard. The BASELINE picture was always accompanied by a picture of the same face with either slightly increased freckles (+25%; PLUS) or slightly decreased freckles (-25%; MINUS) (location left: 720 × 960 pixels [center of the picture], right: 1200 × 960 pixels [center of the picture]) with the spatial position counterbalanced over the whole task. Note that the two presentation positions were different from the adaptation phase, that is, both pictures were presented next to each other in the middle of the screen. Pictures were presented for 1,500 ms, and masks appeared afterward for 300 ms; this was done to avoid afterimages and thus an extended exposure to the visual information of the test stimuli. During the test phase, always three sets of pictures were presented (participants adapted to two sets and participants did not adapt to one set), resulting in a total of 18 faces (note that 12 BASELINE faces were from the learning phase, and six faces were new for usage in the cross-identity condition). The same or corresponding pictures A or B of each set (I, II, III) were presented depending on the image sets during adaptation to apply the three different transfer levels. For the pictorial transfer level, the same picture version was presented as in the adaptation phase (e.g., Pictures A from Set II in both phases). For the structural transfer level, the other picture version was presented (e.g., if Pictures A from Set I were presented during adaptation, Pictures B from Set I were presented during the test phase, and vice versa). For the cross-identity transfer level, images of the set not shown during adaptation were used as test targets (e.g., Pictures A from Set I during the adaptation phase and Pictures A from Set III during the test phase). In total, 72 trials (18 faces × 4 spatial arrangements) were presented.

The entire study, including the learning phase, adaptation phase, break, and test phase, plus the debriefing at the end, lasted about 1.5 hours.

## Results

Results are presented for the learning and test phases.

### Learning phase

On average, participants needed 5.2 (*SD* = 4.2) learning rounds until they could fully correctly name the 12 presented faces, twice upright and inverted. They made 9.5 errors (*SD* = 11.1) on average during the upright learning test and 1.4 errors (*SD* = 1.6) on average during the inverted learning test (note: the inverted learning test only followed if the upright learning test was successfully completed, so measures are not independent and should not be interpreted in a comparative way).

### Test phase

**Figure 3.**
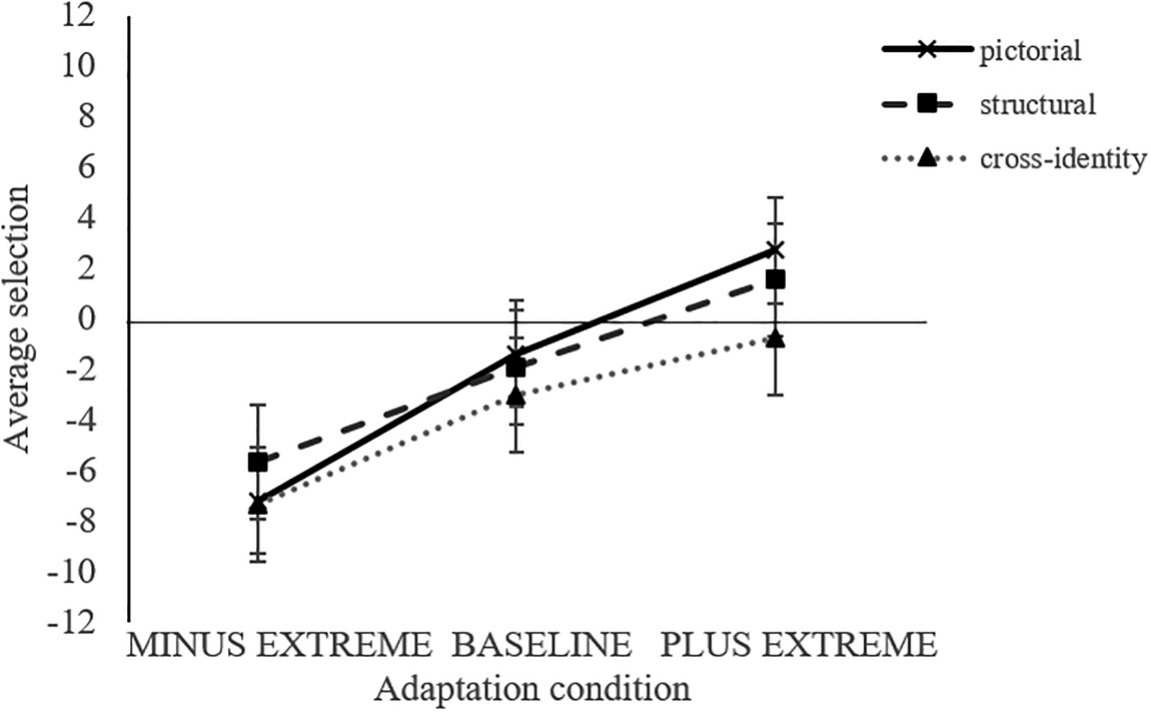
Illustration of the average selection (mean intensity of freckles of the selected test stimulus) for the three adaptation conditions (MINUS EXTREME, BASELINE, and PLUS EXTREME) and the three transfer levels (pictorial, structural, and cross-identity). Error bars represent ±1 standard error of the mean.

Trials with reaction times (RTs) faster than 200 ms and slower than 3 *SD*s above the individual RT mean were excluded from further analyses (resulting in a data loss of 1.7%). Target selection was the dependent variable. It was scored according to the version of the selected face (MINUS = −25; BASELINE = 0; PLUS = +25). A mixed-design ANOVA with the within-participants factor *transfer level* (pictorial, structural, and cross-identity) and the between-participants factor *adaptation condition* (MINUS EXTREME, BASELINE, and PLUS EXTREME) was conducted. With the given test power, we were able to detect a medium-large main effect for adaptation condition, *F*(2,33) = 3.81; *p* = .033; $\eta_{p}^{2}=.187$ . Pairwise comparisons (Bonferroni-corrected) showed a significant difference between the MINUS EXTREME (*M* = −6.74; *SD* = 0.87) and PLUS EXTREME (*M* = 1.16; *SD* = 0.19) adaptation conditions (*p* = .029). There was no difference between BASELINE (*M* = −2.13; *SD* = 1.12) and both EXTREME conditions (BASELINE/MINUS EXTREME: *p *= .355; BASELINE/PLUS EXTREME: *p* = .784). Furthermore, there was a small effect for transfer level, *F*(2,66) = 2.85; *p* = .065; $\eta_{p}^{2}=.079$ , which, however, did not reach the conventionally but arbitrarily pre-set significance level of .05. Pairwise comparisons (Bonferroni-corrected) did not show a significant difference between the three transfer levels (pictorial: *M* = −1.96, *SD* = 8.18; structural: *M* = −2.02, *SD* = 8.12; cross-identity: *M* = −3.72, *SD* = 8.06; all *p*s > .05). We were not able to detect an interaction of both factors, *F*(4,66) = 0.822; *p* = .516; $\eta_{p}^{2}=.047$ . The overall pattern of results can be retrieved from Figure 3.

## Discussion

The present study aimed to further increase knowledge about the role of non-configural face information on adaptation effects. In the studies by Mueller et al. (2021a, 2021b), non-configural face features were manipulated by changing the brightness or saturation of famous faces, and the results showed relatively robust adaptation effects to non-configural face manipulations (at least 5 minutes). These manipulations on famous faces might appear as quite unnatural changes to the faces. Our novel approach employed in the present study was to investigate adaptation effects related to rather *natural* changes in non-configural facial information, which had been relatively unaddressed in research so far.

A prominent feature that changes in many faces on a natural basis seasonally is the intensity of freckles. Our study utilized pre-experimentally unfamiliar faces for which no standard level of freckle intensity could be assumed before the start of the experiment and thus in face representations; therefore, changes might not appear as “irritating” as they might be for famous faces. With this stimulus material and design that are ecologically more valid, we could enhance our understanding of the adaptation to non-configural face features. Despite the advantages of being more ecologically valid, this resulted in a limitation of our study: in previous adaptation studies, famous faces were used, and participants, therefore, had representations of all presented faces. We generated those representations for the faces presented in the learning phase. Obviously we could not generate those representations for the newly presented faces of the cross-identity condition in our test phase. Therefore, there could be a strategy switch of our participants during the presentation of an BASELINE face accompanied by a new (cross-identity) face. There is a small descriptive difference in response pattern for the cross-identity condition between BASELINE and PLUS EXTREME condition. This could be a small hint for an actually slightly different response pattern for the cross-identity condition. However, we did not find statistical differences for the cross-identity condition compared to the pictorial or structural transfer conditions. The adaptation effect found here is probably not the result of a strategy switch, but of a memory-based empirical norm such as a “reference” or “prototype” established by the participants during the adaptation phase. Furthermore, other adaptation studies using both familiar and unfamiliar faces could detect adaptation effects not only to familiar or famous faces, but also to unfamiliar ones (e.g., Ewbank & Andrews, 2008; Ewbank et al., 2008). In previous adaptation studies by our group and our more recent studies (e.g., Mueller et al., 2021a, 2021b), we could show that where adaptation took place, those effects transferred to other not previously seen faces (our cross-identity condition). Therefore, the effect found in the cross-identity condition in our present study seems to be in line with previous adaptation studies and to be actually the result of changes in our whole face space (our “experience” space), where the last faces we have seen have an especially strong influence.

By using a paradigm that separated the adaptation and test phases by approximately 5 minutes (see Carbon, Strobach, et al., 2007), we could test if those adaptation effects are not only based on short-term perceptual processes but are more representationally related. We observed clear adaptation effects (with a medium-large effect size), which show robustness even after a 5-minute break. Furthermore, since the positions from the adaptors (six different positions: top-left, bottom-left, top-middle, bottom-middle, top-right, bottom-right) and from the test pictures (two presented in the middle of the screen) did not overlap, we can conclude that our results cannot simply be explained by retinotopic effects (i.e., a change in the perception of the actual image). Although we cannot rule out the possibility that there might be a retinal effect, we presented some evidence pointing in the direction of a memory effect (i.e., change in the memory representations of the BASELINE faces).

We did not find significant differences between the baseline and both extreme levels. On a descriptive level, it also looks like a linear effect and that both extreme groups are in fact different from the BASELINE condition. However, this did not reach significance. This lack of effect could be due to more subtle shifts in mental representations, that is, shifts smaller than +25%, that could not be detected with the design used in the present study. Future studies should investigate this possibility by using a response format with more alternatives to choose from, that is, faces with different and finer-graded freckle intensities.

The adaptation effects showed up across all transfer levels (pictorial, structural, and cross-identity), indicating that the uncovered effects were not bound to specific pictorial depictions but generalized across different depictions and even other faces similarly. Other studies addressing configural face adaptation effects found weaker adaptation effects on the structural level compared to the pictorial level (e.g., Carbon & Ditye, 2012; Carbon & Leder, 2005b) and also weaker effects for the cross-identity level in comparison to both other levels (e.g., Carbon & Ditye, 2011, 2012; Carbon, Strobach, et al., 2007; Strobach et al., 2011; review by Mueller et al., 2020). This indicates that exposing people to faces of certain levels of freckle intensities lets them adapt to general levels of freckle intensities independently from specific face exemplars, for instance, the ones that the participants were familiarized with. In terms of the theory of face space (Valentine, 1999), this would mean that the whole face space changes toward the conditions of previously experienced facial depictions (in our case, the intensity of freckles). With regard to the overall strength of our adaptation effect (about 5%), the effect found in the present study is rather weak in comparison to 20% by Carbon, Strobach, et al. (2007) and 25% in Webster and MacLin (1999), but similar to Leopold et al. (2001) with 5%.

Since the adaptation effect found in the present study was general (not identity specific), the question arises if our effect is even a face-specific effect. In our paper by Mueller et al. (2021a), we could find evidence that brightness manipulations are face-specific through two additional experiments: we used scrambled faces (i.e., each image was divided into very small pieces and randomly assembled so that one homogeneous color area was created, representing the average color of the respective adaptation image) and inverted faces (same faces differing only in orientation, i.e., rotated by 180 degrees). So, there is some evidence that facial information is necessary for our brightness adaptation effects, but we did not test this so far explicitly for freckles. Both experiments would therefore be necessary experiments for future studies to clarify the face-specificity of adaptation to freckles. If our effects are face-specific, than holistic processing of faces should be necessary. If this is disturbed by inversion or by scrambling faces, results should show no adaptation effects. Then we can conclude that adaptation to freckles is not simply the result of adaptation to color or patterns, but that facial information is necessary. Future research could also address face-specificity by using, for example, face-like forms (e.g., simple ovals) filled with dots that are similar to the freckles used in our study, but without facial features. If our results are not face-specific, results should look similar to the results presented here. If this is the case, it could also be explained by the texture density adaptation effect shown by MacKay (1964) during adaptation to sandpaper or random dots. Anstis (1974) suggests that this is the result of adaptation to the spatial frequencies of the textures and gratings used in their study. Durgin and Huk (1997) could show that adaptation to density differences in artificial textures leads to density aftereffects in naturalistic textures if spatial frequencies are held constant. Therefore, the spatial frequency of our freckle pattern would be the crucial point for adaptation, not the fact that we presented freckled faces.

Despite the shortcomings of adaptation paradigms in general, we can conclude that there are not only relatively robust adaptation effects to non-configural features using quite unnatural changes in features of famous faces but also more natural changes to pre-experimentally unfamiliar faces. The perception of freckles seems to be considerably adaptive, which might be the reason that in most situations, we do not recognize them at all. This neglect of perceiving them consciously might change in Umwelten that does not provide faces with freckles, such as in specific geographic regions or during seasons where freckles are less prevalent. The results are also in line with our experience in spring, when we detect those relatively salient changes in familiar faces (the appearance of freckles), but stop registering them after only a few exposures due to a loss of information quality.

## Notes

1. The exact wording of the relevant part of the instruction was the following: “For the following task, you will always see two faces presented next to each other. Your task is to decide if the left or the right picture is the “baseline” face. The “baseline” face means the face you have seen in the first section of the experiment. Remember, that during this section, your task was to learn the names of the depicted faces.”
